# Comparative genomics of Shiga toxin-producing *Escherichia coli* reveals host-specific adhesiome adaptations in humans and cattle

**DOI:** 10.3389/fvets.2025.1639243

**Published:** 2025-10-09

**Authors:** Víctor Martínez, José T. Cartajena, Estefanía Méndez, Jessica Dörner, Diego Méndez, Gabriel Arriagada, Jorge Toledo, Richard Arancibia, Nicolás Pizarro, Daniela Castro, Daniela Luna, Romina Ramos, Joaquín Jorquera, Beatriz Escobar, Indira T. Kudva, Nicolás Galarce

**Affiliations:** ^1^Departamento de Ciencia Animal, Facultad de Ciencias Veterinarias y Pecuarias, Universidad de Chile, Santiago, Chile; ^2^Departamento de Medicina Preventiva Animal, Facultad de Ciencias Veterinarias y Pecuarias, Universidad de Chile, Santiago, Chile; ^3^Programa de Magíster en Ciencias Animales y Veterinarias, Facultad de Ciencias Veterinarias y Pecuarias, Universidad de Chile, Santiago, Chile; ^4^Life Sciences Faculty, Universidad Andres Bello, Santiago, Chile; ^5^Instituto de Ciencias Agroalimentarias, Animales y Ambientales, Universidad de O’Higgins, San Fernando, Chile; ^6^Red de Equipamiento Científico Avanzado, Facultad de Medicina, Universidad de Chile, Santiago, Chile; ^7^Departamento de Ciencias de la Salud, Universidad de Aysén, Coyhaique, Chile; ^8^Departamento de Ciencias Clínicas, Facultad de Ciencias Veterinarias y Pecuarias, Universidad de Chile, Santiago, Chile; ^9^Instituto de Investigaciones Agropecuarias, Osorno, Chile; ^10^Facultad de Medicina Veterinaria y Agronomía, Universidad de las Américas, Santiago, Chile; ^11^Escuela de Medicina Veterinaria, Facultad de Ciencias Agrarias y Forestales, Universidad Católica del Maule, Curicó, Chile; ^12^Escuela de Medicina Veterinaria, Facultad de Recursos Naturales y Medicina Veterinaria, Universidad Santo Tomás, Santiago, Chile; ^13^Escuela de Medicina Veterinaria, Facultad de Ciencias de la Vida, Universidad Andrés Bello, Santiago, Chile; ^14^Food Safety and Enteric Pathogens Research Unit, National Animal Disease Center, Agricultural Research Service, United States Department of Agriculture, Ames, IA, United States

**Keywords:** STEC, *E. coli*, Shiga, adhesiome, WGS, GWAS, cattle, human

## Abstract

**Introduction:**

Shiga toxin-producing *Escherichia coli* (STEC) is a zoonotic pathogen responsible for severe human infections, with cattle recognized as the principal animal reservoir for human infection. Adhesion is a critical step in STEC colonization, facilitating persistence and transmission. While human-associated adhesion mechanisms have been extensively studied, those driving colonization in cattle remain less understood. In this study, we characterized the adhesiome of STEC strains isolated from Chilean cattle and compared them with a global collection to identify host-specific adhesion patterns and genetic adaptations.

**Methods:**

A total of 948 fecal samples from Chilean cattle were screened, yielding 71 confirmed STEC isolates, which were analyzed alongside 546 publicly available genomes to compare host-specific adhesion patterns. The adhesiome was examined based on gene presence/absence patterns, followed by a genome-wide association study (GWAS) and variant effect analysis to identify host-specific adhesion genes and their functional implications.

**Results:**

Adhesin gene analysis revealed distinct adhesion strategies between hosts. Several genes, including *ehaA*, *stgABC*, *yadLMN*, and *iha*, were significantly associated with cattle, while *eae*, *cah*, *ypjA*, and *paa* were more frequent in human-associated STEC. Functional enrichment analysis revealed differences in biological processes, including protein folding and fimbrial usher porin activity in cattle, and response to methylglyoxal in humans. GWAS identified *yeeJ*, *espP*, and *fimC* as strongly associated with cattle strains, whereas *clpV*, *ybgQ*, and sab were linked to human isolates. Variant analysis showed higher genetic diversity in human isolates, with *yadK*, *espP*, and *ybgP* exhibiting the highest variant densities. However, the functional effects of adhesin mutations were largely conserved across hosts, suggesting selective constraints on adhesion mechanisms.

**Discussion:**

Our findings provide new insights into STEC host adaptation and highlight potential targets to reduce zoonotic transmission and improve pre-harvest food safety strategies. Future research should focus on functional validation of host-specific adhesin variants and their potential as preventive strategies.

## 1 Introduction

Shiga toxin-producing *Escherichia coli* (STEC) are a group of emerging zoonotic pathogens responsible for significant public health and economic burdens worldwide (1). These bacteria produce Shiga toxins (Stx), potent cytotoxins that can cause severe human diseases, including hemorrhagic colitis and hemolytic-uremic syndrome (HUS), especially in young children (2). STEC is primarily transmitted to humans through consumption of contaminated food, particularly beef (3).

Adult cattle are recognized as the primary reservoir of STEC, with reported prevalence rates in Latin America ranging from 14 to 90% (4, 5). Their widespread presence increases the risk of environmental contamination and zoonotic transmission, underscoring the need for effective livestock-based control strategies. Beyond human health concerns, STEC infections impose substantial economic costs related to healthcare, productivity loss, and outbreak management. In the United States alone, healthcare costs associated with STEC infections were estimated to exceed USD 311 million in 2018 (6).

STEC utilizes diverse adhesins—including surface-associated and secreted proteins—to colonize host tissues (7). A key adhesion determinant is the locus of enterocyte effacement (LEE), which encodes intimin (*eae*) and type III secretion system (T3SS) proteins, promoting intimate attachment to enterocytes and microvilli effacement (8). LEE-positive strains, such as O157:H7 and several non-O157 serotypes (e.g., O111:NM, O26:H11, O103:H2), are strongly associated with outbreaks and severe human disease (9). However, the emergence of LEE-negative STEC strains utilizing alternative adhesins, encoded in pathogenicity islands like the locus of adhesion and autoaggregation (LAA) and the locus of proteolysis activity (LPA), highlights additional colonization strategies (10, 11). Collectively, these adhesion determinants constitute the adhesiome, defined as the complete set of fimbrial and non-fimbrial adhesin genes that facilitate bacterial colonization of host tissues (12).

While human-associated adhesion mechanisms have been extensively characterized, those facilitating bovine colonization remain poorly understood. Since cattle serve as the main reservoir for human infection, elucidating these mechanisms is critical for designing targeted pre-harvest interventions. Newborn calves are typically colonized by STEC shortly after birth, acquiring the pathogen from maternal microbiota and the surrounding environment (13, 14). Once ingested, STEC can survive, persist, and colonize the gastrointestinal tract, particularly targeting the recto-anal junction (RAJ) as its primary site of colonization (15, 16). It has been shown that proteins encoded by the LEE play a critical role in STEC adherence to RAJ’s stratified squamous epithelium (RSE) cells, including intimin (17, 18). However, other adhesins are involved in the colonization and persistence of LEE-positive STEC in cattle, such as EhaA (19), and H7 flagella (20), among others. Moreover, Kudva et al. (21) registered a similar LEE-positive adhesion pattern of LEE-negative STEC strains to RAJ cells, concluding that adhesins other than intimin are involved in this phenotype. Adhesion of STEC to bovine gut by specific virulence factors is of paramount importance since it allows its persistence and the successful transmission to other hosts (22). Therefore, understanding STEC colonization is the key step to controlling the infection.

The genetic diversity of STEC, including variations within the adhesiome, poses a major challenge for prevention strategies, such as vaccine development. The complexity of STEC adhesion mechanisms across hosts underscores the need for genomic surveillance to characterize circulating strains and their colonization traits (23, 24). This study aims to characterize the adhesiome of STEC strains isolated from cattle in Chile and compare them with global strains to identify host-specific adhesion patterns. Understanding these colonization mechanisms is fundamental for designing effective intervention strategies to mitigate STEC transmission at the livestock level. By elucidating key adhesins involved in bovine persistence, this research contributes to the development of targeted mitigation strategies, contributing to One Health-based strategies to mitigate transmission risks at the human-animal interface.

## 2 Materials and methods

### 2.1 Sample collection for STEC isolation

A total of 948 fecal samples were collected between June 2023 and March 2024 from abattoirs and farms located across seven regions, representing the majority of Chile’s cattle population (25). The sampled animals included both juveniles and adults, with most being of mixed breed. Approximately 20 g of fecal material per animal was aseptically collected directly from the rectum by trained veterinarians and transported under refrigerated conditions in sterile flasks until laboratory processing. All sampling procedures were approved by the Institutional Committee of Care and Use of Animals, Universidad de Chile (Protocol No. 23658—VET—UCH).

### 2.2 Sample processing and STEC identification

Samples were processed following protocols from previous studies (26, 27). Briefly, 5 g of each fecal sample were enriched in 9 mL of tryptone soy broth (Becton Dickinson and Co., United States) and incubated overnight at 42 °C. A 25 μL aliquot of the enrichment culture was then plated onto MacConkey agar (Becton Dickinson and Co., United States) and incubated at 37 °C for 18–24 h.

Bacterial growth from confluent areas was resuspended in 500 μL of sterile nuclease-free water, subjected to heat treatment at 100 °C for 15 min, and centrifuged at 26,480 × g for 5 min. DNA concentration and purity (A260/A280 ratio) were determined using a NANO-400 micro-spectrophotometer (Hangzhou Allsheng Instruments Co., China). Samples with optimal purity (1.8–2.0) were stored at −20 °C for subsequent analyses.

The presence of *stx1* and *stx2* genes was confirmed by multiplex PCR (28) in a LifeECO^®^ thermal cycler (Hangzhou Bioer Technology Co., China). The STEC97 strain [*stx1*-positive, *eae*-positive, *stx2*-positive (29)] was used as a positive control, while *E. coli* ATCC 25922 served as the negative control. Up to 30 colonies per positive sample were individually plated on MacConkey agar (Becton Dickinson and Co., United States) and CHROMagar^™^ STEC (CHROMagar Microbiology, France) (30, 31). After 24 h of incubation, colonies were screened by multiplex PCR to confirm the presence of *stx1* and/or *stx2* genes. PCR-confirmed colonies were then tested for the *uspA* gene, encoding the universal stress protein A, to verify *E. coli* species identity (32). A single confirmed isolate per sample was stored at −80 °C for further analysis.

### 2.3 Whole genome sequencing of STEC strains

Genomic DNA from all STEC strains was extracted using the Wizard Genomic DNA Purification Kit (Promega, United States), following the manufacturer’s instructions. DNA concentration was measured using fluorometry with the Qubit^®^ dsDNA BR Assay kit (Life Technologies, United States), and DNA quality was assessed with an Epoch microplate spectrophotometer (Biotek Instruments, United States). A total of 1 ng of DNA was used for library preparation using the Nextera XT DNA Library Prep Kit (Illumina, United States), following the manufacturer’s protocol. The average fragment size of libraries was determined by capillary electrophoresis using the High Sensitivity NGS Fragment Analysis Kit (Advanced Analytical Technologies, United States). Libraries were quantified using the KAPA Library Quantification Kit (Kapa Biosystems, United States) on a Rotor-Gene Q platform (Qiagen, Germany). Whole-genome sequencing (WGS) was performed on a NovaSeq X Plus platform (Illumina, United States) with a 150-cycle paired-end reagent kit at Haplox Company, Hong Kong.

Additionally, two STEC strains isolated from clinical human stool samples, corresponding to serotypes O157:H7 and O26:H11 and provided by the Instituto de Salud Pública de Chile, were included in the analysis for comparison purposes. These strains were processed for WGS as described above. All genome sequences were deposited in GenBank under BioProject number PRJNA656305.

### 2.4 Publicly available sequence data

To enhance the comparative analysis, 568 publicly available *E. coli* genome sequences were retrieved from GenBank’s Sequence Read Archive (SRA)^1^ on December 15, 2024. Genomes were selected based on diverse geographical origins, host species, and serotypes to broadly represent the global diversity of STEC strains.

All genomes were screened for the presence of *stx* subtypes by mapping reads against reference sequences using BWA (33). After filtering, a final dataset comprising 546 confirmed STEC genomes was obtained for downstream analysis. Metadata, including host origin, country of isolation, and year, were retrieved using the eSearch tool from the EMBOSS suite (34).

### 2.5 Epidemiological typing and phylogenomic analysis

All FASTQ Illumina reads were assembled *de novo* using SPAdes (v.3.15.2) with default parameters (35). Genome assembly quality was evaluated using CheckM2 (36), which estimates genome completeness and contamination based on machine learning models trained with lineage-specific marker sets available in the DIAMOND^2^ database.

The program was not run using a specific model; a cosine similarity calculation was performed to determine the appropriate completeness model for each isolate. The program predicts protein sequences to annotate all the genomes with DIAMOND. Finally, in all cases the Neural network contamination model was used.^3^ This quality control step was critical to ensure the reliability of assemblies, particularly for genomes retrieved from the SRA. Only genomes with a completeness score greater than 99.6%, as estimated by CheckM2, were included in the analysis.

Prediction of *stx* subtypes was performed on ABRicate (v.0.8.13).^4^ Sequence types (STs) of all STEC strains were predicted by Achtman’s multilocus sequence typing (MLST) scheme using the GitHub platform.^5^ The housekeeping genes used included *adk*, *fumC*, *gyrB*, *icd*, *mdh*, *purA*, and *recA*. Additionally, SerotypeFinder 2.0^6^ was used to determine serotype (37).

### 2.6 Adhesiome analysis

Adhesin gene analysis was conducted using AdhesiomeR,^7^ an R-based tool that executes BLASTn searches against a curated database of 427 adhesin genes identified across diverse *E. coli* strains. Adhesin sequences were classified according to sequence identity thresholds: highly similar (>95%), moderately similar (75–95%), and unrelated (<75%) (12). In order to enhance confidence in adhesin identification, a strict mode with gene-specific bit score threshold was applied. Comparative analyses were based on presence/absence matrices (1 = present; 0 = absent) and clustering profiles to detect host-specific adhesin signatures between human- and cattle-associated STEC isolates. Differences in the detection rates of adhesin genes between cattle- and human-associated isolates were analyzed using a *Z*-test for two proportions. The analysis was performed in Microsoft Excel (Microsoft Office 365, version 16.100.2), with statistical significance set at *p* < 0.05.

To further explore genetic variability, we performed genome-wide association analysis (GWAS) using two complementary approaches. First, we used the complete genome of *E. coli* K-12 (ASM584v2) as the reference, which contains the majority of core adhesin genes and allowed variant calling and functional annotation at the whole-genome level. Second, recognizing that several adhesin genes were absent from the K-12 genome, we conducted an additional GWAS focused on the adhesiome. For this, we extracted all 427 adhesin gene sequences identified in our adhesiome analysis—including those shared between human- and cattle-associated strains—to ensure a more comprehensive assessment. These sequences were retrieved from AdhesiomeR^8^ and used as templates for further analysis. The complete genome sequences of STEC strains isolated from stool samples (*n* = 158 from cattle and *n* = 205 from humans) were mapped to the *E. coli* K-12 genome and to the adhesiome sequences using BWA (38). After sorting using BamTools (39), the PCR duplicates were removed using Sambamba (40). Single nucleotide polymorphisms (SNPs) and short insertions/deletions (indels) were identified using FreeBayes and jointly using all the samples in the analysis (41).

### 2.7 Genome-wide association analysis

GWAS was conducted based on SNPs and indels identified from variant call format (VCF) files generated by FreeBayes. Quality control of variants was performed using VCFtools, applying a minimum quality threshold (MinQ) of 30 and a mean depth filter of 100× (42). A logistic regression model was fitted to the data, using host species (cattle = 1, human = 0) as the binary outcome, following the approach described by Pérez-Enciso et al. (43).

We used Pyseer (44) to discover variants significantly associated with the cattle host, being humans deemed as controls in the standard association analysis. To account for potential confounding by population structure, we conducted a principal component analysis (PCA) on variant call format (VCF) files using PLINK [-pca command (45)]. The model included the first five principal components as fixed effects, as eigenvalues showed no substantial drop beyond this threshold. The pairwise distance matrix was generated using Mash (46). Following the Benjamini and Hochberg correction, the *p*-values were adjusted for multiple comparisons using the false discovery rate (FDR) method, implemented through the p.adjust function in R (FDR ≤0.05). Model coefficients were also presented as odds ratios (OR), with values greater than one indicating an association of the alternative allele with the cattle host.

Annotation and functional impact prediction of host-associated variants were performed using snpEff (47). For GWAS analyses based on the *E. coli* K-12 genome, the pre-compiled *E. coli* str. K-12 substr. MG1655 database provided by SnpEff was employed. For the adhesiome sequences that belonged to different bacterial strains, we constructed a custom SNPeff database. The adhesiome sequences were annotated using AUGUSTUS (48), with the *E. coli* K-12 database selected as the reference strain to guide the annotation (obtained as a GFF file format). This annotation file was then converted to GTF format using AGAT to develop the snpEff custom database of the adhesiome. Predicted coding sequences (CDS) and protein sequences of the identified genes were obtained using the script getAnnoFasta.pl (available at https://github.com/nextgenusfs/augustus.git). All these files were used to compile the snpEff database.

## 3 Results

### 3.1 STEC identification in Chilean cattle

**Figure 1:**
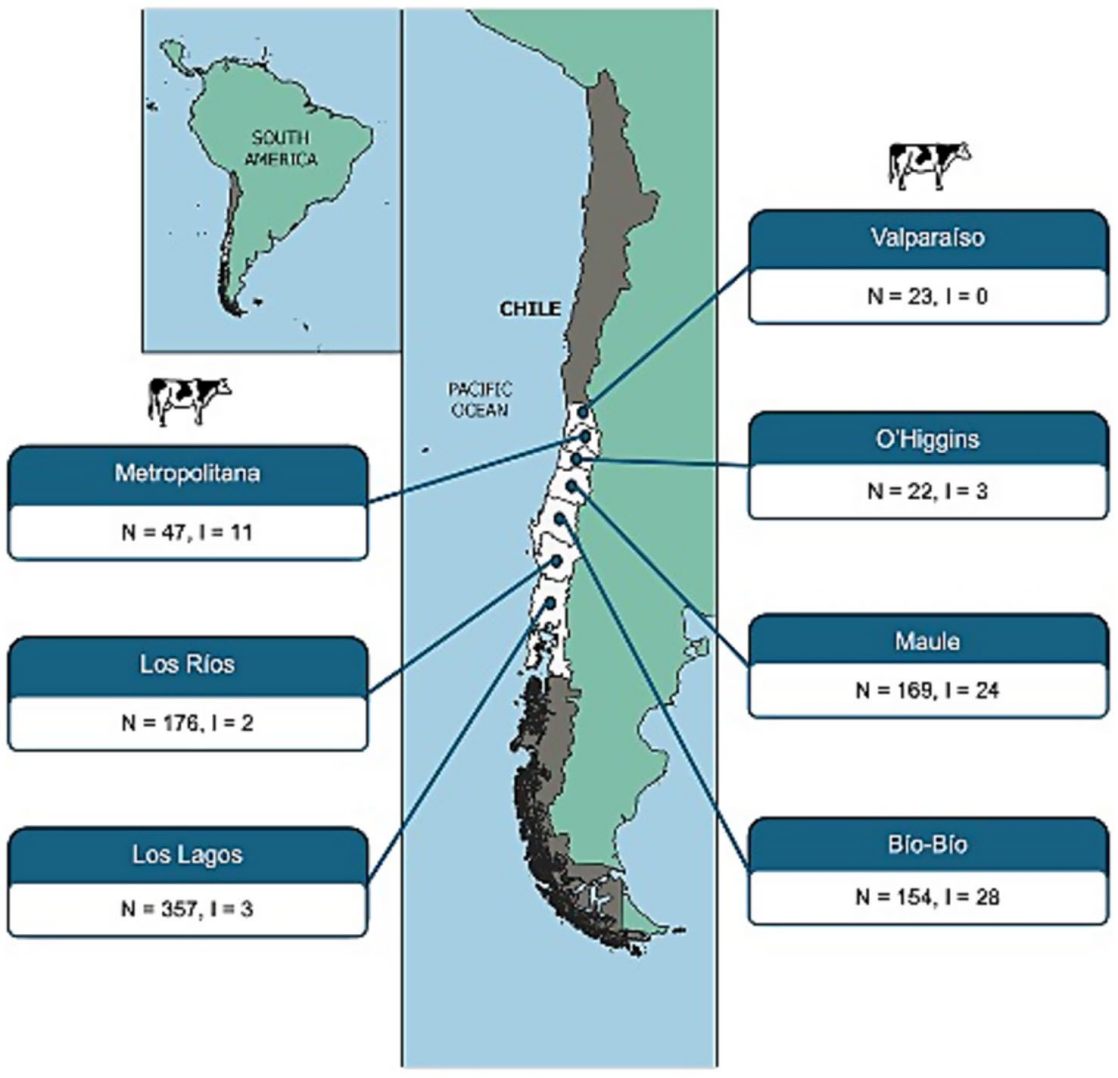
Geographical distribution of STEC strains isolated from cattle feces across Chilean regions, based on official administrative divisions. For each region, the number of fecal samples collected (N) and the number of STEC-positive isolates recovered (I) are indicated.

Of the 948 fecal samples collected from cattle, 71 (7.5%) were confirmed as STEC-positive, harboring *stx1* and/or *stx2* genes along with the *uspA* marker. Among these, 70.4% harbored *stx2*, 26.8% carried both *stx1* and *stx2*, and only 2.8% were positive for *stx1* alone. The geographical distribution of STEC varied across Chile, with regional differences in detection rates (Figure 1).

### 3.2 Epidemiological typing and phylogenomic analysis

For comparative purposes, 546 publicly available STEC genomes were retrieved from NCBI, encompassing isolates from humans (50.0%), food (23.8%), and cattle (19.4%). These genomes spanned a broad temporal range (1978–2021) and were predominantly collected from Germany (43.8%), Chile (16.1%), and France (13.2%).

**Figure 2:**
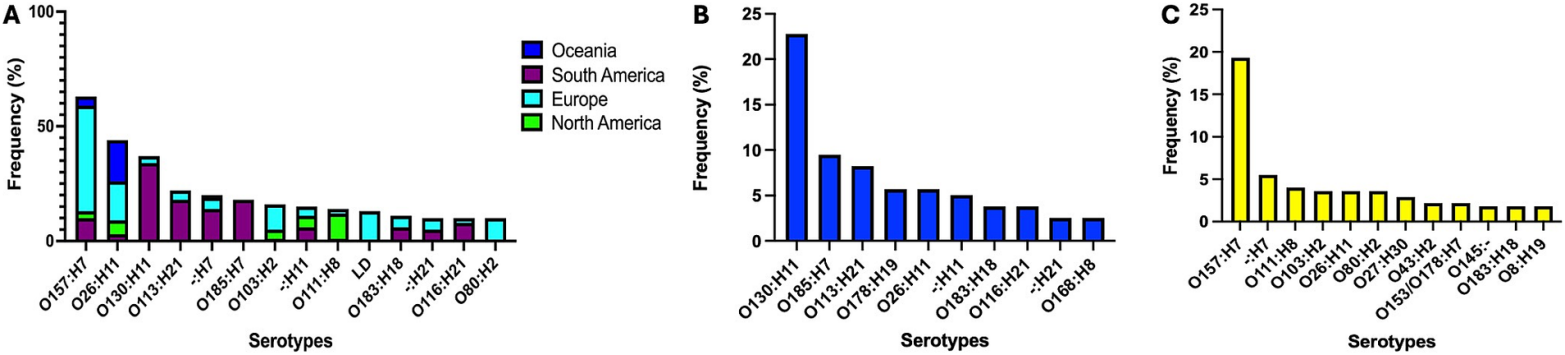
Distribution of major STEC serotypes across continents **(A)** and by host species (**B**, cattle; **C**, humans). Selected continents represent 96.7% of analyzed genomes. LD, low discrimination between predicted serotypes.

**Figure 3:**
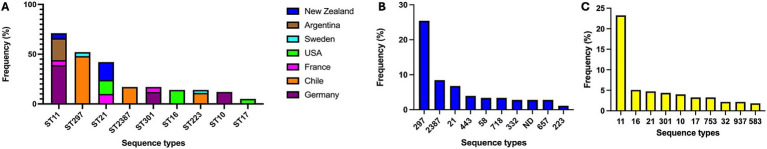
Distribution of the most frequent STEC sequence types (STs) across countries **(A)** and host species (**B**, cattle; **C**, humans). Selected countries represent 96.7% of analyzed genomes.

Among the 619 genomes analyzed (71 Chilean isolates plus 546 public genomes), we identified 29 distinct *stx* subtypes profiles, with *stx2a* (20.0%) and *stx1a* (18.9%) being the most prevalent. Serotype analysis indicated O157:H7 (10.8%), O26:H11 (7.1%), and O130:H11 (6.0%) as the most frequently detected. MLST classification identified 141 STs, with ST11 (13.4%) and ST297 (8.7%) being the most common. Complete metadata is provided in Supplementary Table 1. Figures 2, 3 depict the distribution of serotypes and STs according to geographical origin and host.

Among the 71 STEC strains isolated in this study, eight *stx* subtypes profiles were identified, with *stx2d* (38.0%), *stx2c* (26.8%), and *stx1a* + *stx2a* (12.7%) being the most prevalent. Among the 12 predicted serotypes, O130:H11 (42.3%), O185:H7 (21.1%), and O113:H21 (8.5%) were the most common. Likewise, the most frequently detected STs were ST297 (49.3%), ST2387 (20.5%), and ST223 (9.6%).

**Figure 4:**
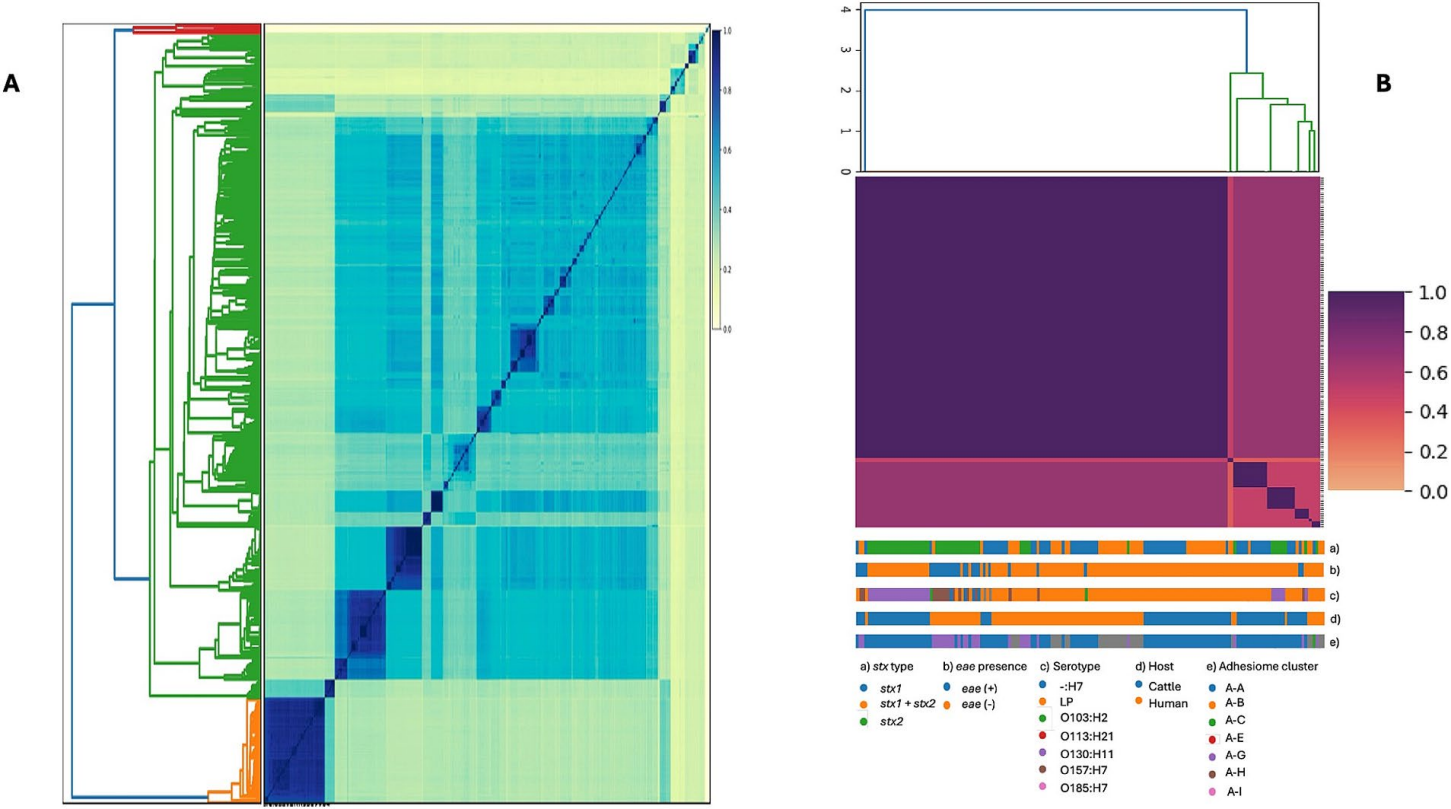
Genome similarity among STEC isolates based on whole-genome sequences. **(A)** Pairwise genome similarity (Jaccard similarity index, JSI) among 572 isolates from cattle, humans, and food. **(B)** Genetic diversity of the subset of isolates included in the adhesiome analysis, restricted to human and cattle stool samples. Colored tracks indicate *stx* type (a), *eae* presence (b), serotype (c), host (d), and adhesiome cluster (e). In both panels, darker colors represent higher similarity (JSI close to 1).

Due to the high diversity in serotypes, *stx* profiles, and STs, we examined the genomic structure of STEC isolates from food, cattle, and humans (*n* = 572) to assess whether genomic similarity clusters corresponded to host origin or geographic region. Based on STs, we generated MinHash sketches of draft whole-genome assemblies using k-mers of length 31 and a sketch size of 100,000 in Sourmash. This analysis revealed substantial genomic variability between groups, potentially enhancing STEC adaptability to diverse environments and supporting a wide range of virulence strategies (Figure 4). In parallel, genetically homogeneous clades were observed within specific STEC STs, suggesting associations with ecological factors or adaptive niches, further supported by the multidimensional scaling analysis (Supplementary Figure 1).

### 3.3 Adhesiome analysis

#### 3.3.1 Analysis of the pangenome of the adhesiome in host related strains

**Figure 5:**
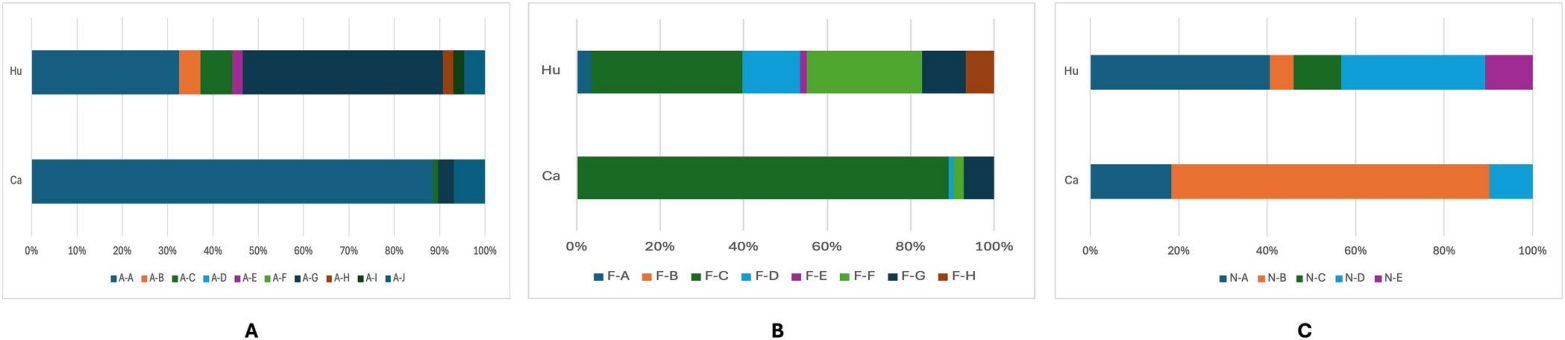
Frequency of adhesin **(A)**, fimbrial **(B)**, and non-fimbrial **(C)** clusters among STEC genomes from humans (Hu) and cattle (Ca). Bars represent the number of genomes identified in each cluster. Different letter combinations denote distinct clusters, following the AdhesiomeR nomenclature.

To characterize the adhesiome of STEC genomes from cattle and human origins, we used the AdhesiomeR tool to identify adhesin clusters, which defined fimbrial and non-fimbrial adhesins function. The distribution of adhesin-related gene clusters among STEC genomes is presented in Figure 5. Adhesin clusters (Figure 5A) revealed a predominance of the A-A cluster among cattle-associated strains (88.5%), while human-associated strains exhibited greater diversity, with A-G as the most prevalent cluster (44.2%). Fimbrial clusters (Figure 5B) were predominantly represented by the F-C cluster in cattle (83.9%), whereas human-associated strains showed a broader distribution, with F-C and F-F being the most frequent (24.4 and 18.6%, respectively). Similarly, among non-fimbrial clusters (Figure 5C), N-B predominated in cattle strains (67.8%), while human strains were more diverse, with higher proportions of N-A and N-D clusters (17.4 and 14%, respectively).

These findings suggest that cattle-associated STEC strains exhibit a more conserved adhesin profile, whereas human-associated strains display greater diversity, possibly reflecting adaptation to host-specific selective pressures and the diversity of the strains analyzed.

**Figure 6:**
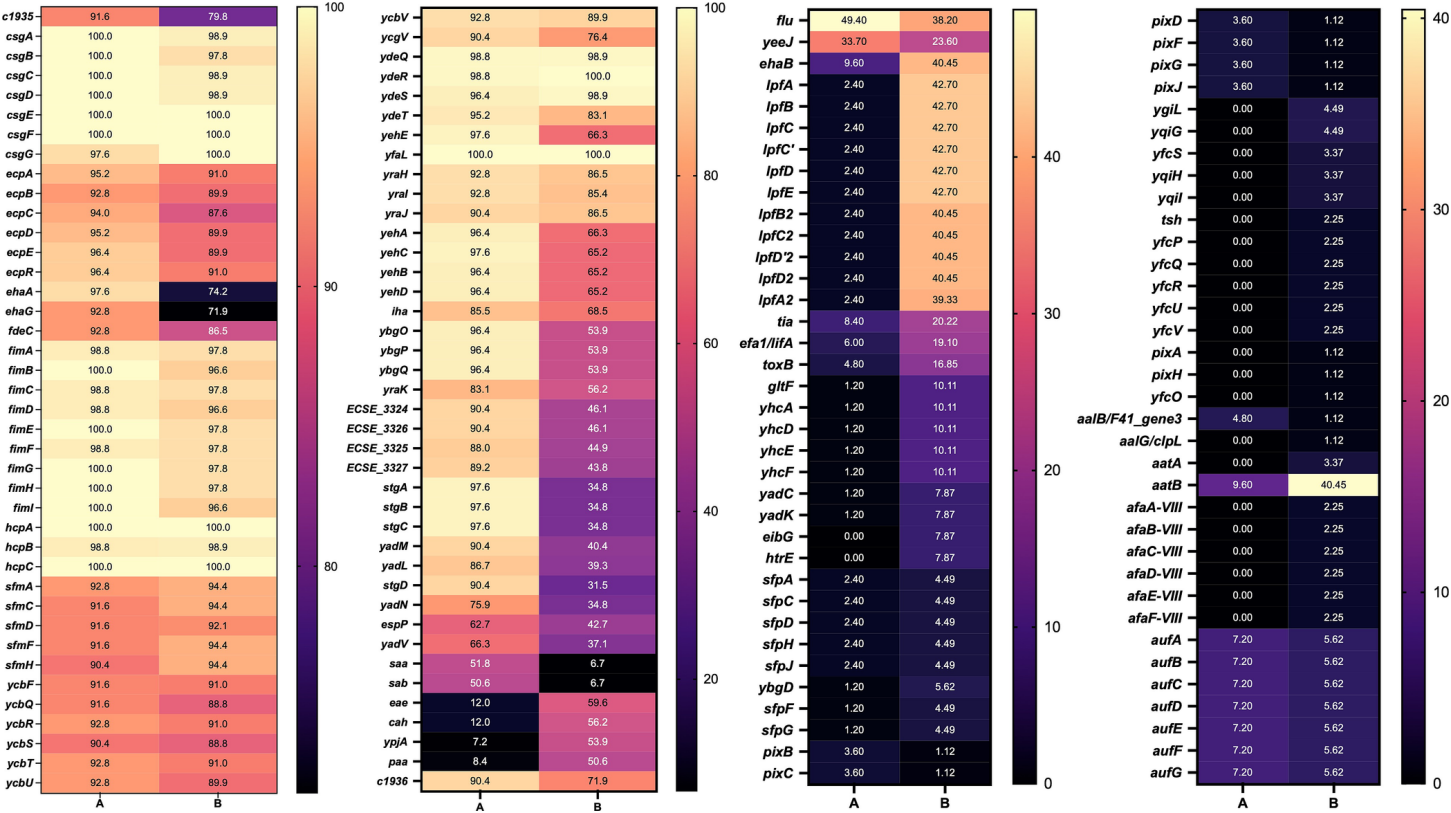
Heatmap of genes present in STEC genomes from cattle and human sources. A: cattle; B: human, numbers indicate the percent prevalence of individual genes.

Next, we compared the genes identified in the adhesin clusters according to their host (Figure 6). This analysis revealed a combination of conserved core adhesins and host-associated adhesion profiles. Many adhesin genes, particularly those encoded within the *fim*, *csg*, and *ecp* operons, were highly conserved across cattle- and human-associated strains (>87% prevalence), indicating that certain adhesion mechanisms are fundamental for bacterial colonization, irrespective of the host species.

Nevertheless, differences were observed for specific adhesins. For example, *ehaA* was more prevalent in cattle (97.6%) compared to humans (74.2%). Similarly, *stgA*, *stgB*, and *stgC* were more common in cattle-associated strains, suggesting a greater reliance on alternative fimbrial adhesins for colonization. Additionally, the *yadM*, *yadL*, *yadN*, and *iha* genes were detected more frequently in cattle strains, supporting their potential role in host-specific adaptation. Conversely, STEC strains from humans showed a higher frequency of *eae*, *cah*, *ypjA*, and *paa*, all of which have been implicated in epithelial attachment and virulence.

Using the gene presence/absence data from the adhesiome, we conducted a *Z*-test for differences in proportions to identify host-specific functional enrichments (Supplementary Table 2). Several biological processes related to pilus biology, including pilus formation (GO:0009289), pilus assembly (GO:0009297), and pilus organization (GO:0043711), as well as cell adhesion involved in single-species biofilm formation (GO:0043709), were significantly enriched in both cattle- and human-associated strains.

In cattle, processes such as protein folding (GO:0006457) and fimbrial usher porin activity (GO:0015473) were significantly enriched. In contrast, response to methylglyoxal (GO:0051595) was significantly enriched in humans (Supplementary Table 3). These findings support the hypothesis that STEC strains exhibit host-specific adhesion strategies, with cattle-associated strains relying more on fimbrial and curli-mediated mechanisms, while human-associated strains may have evolved broader stress response capabilities to persist within the human gastrointestinal environment.

#### 3.3.2 Genome wide association analysis using *Escherichia coli* K-12

**Figure 7:**
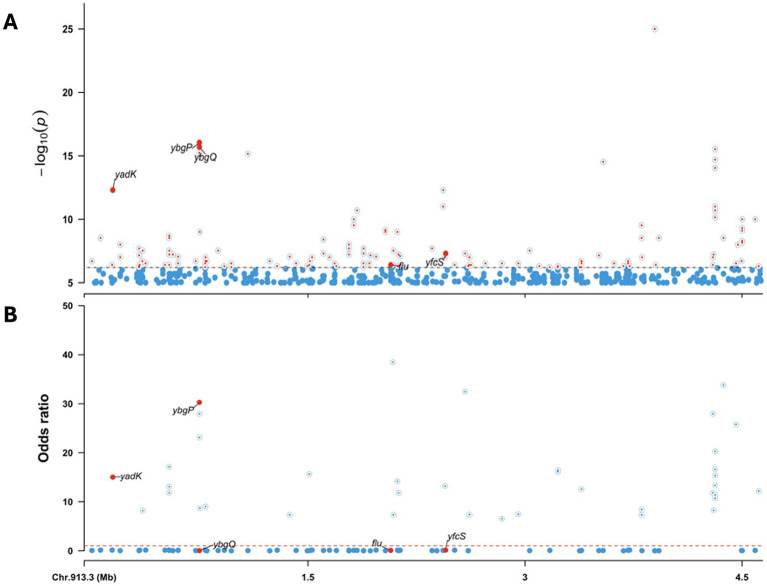
Genome-wide association analysis (GWAS) of human- and cattle-associated STEC strains using the *E. coli* K-12 genome as reference. **(A)** Manhattan plot showing the -log10(*p*) values from the association analysis. Significant variants in the adhesiome (e.g., *yadK*, *ybgP*, *ybgQ*, *flu*, *yfcS*) are indicated by large red dots. **(B)** Odds ratios (OR) derived from regression coefficients, with OR > 1 indicating association with cattle strains and OR < 1 indicating association with human strains. The dashed line at OR = 1 marks the neutral threshold. Together, these results highlight adhesiome genes significantly associated with host specificity.

The GWAS using the *E. coli* K-12 reference genome is presented in Figure 7A. Gene enrichment analysis revealed that cattle-associated genes were significantly linked to trehalose transport (GO:0015771) and protein-phosphocysteine-trehalose phosphotransferase system transporter activity (GO:0090589), involving the *bglF* and *ascF* genes. A complete list of enriched genes and associated pathways is provided in Supplementary Table 4.

Significant variants were predominantly associated with adhesin genes. Genes such as *yadK*, *ybgP*, *yfcS*, *flu*, and *ybgQ* exhibited association signals surpassing the significance thresholds at the whole-genome level. ORs derived from regression coefficients indicated stronger associations of *yadK* and *ybgP* with cattle isolates, while *flu*, *yfcS*, and *ybgQ* were more closely linked to human-associated strains (Figure 7B).

#### 3.3.3 Genome wide association analysis using the adhesiome

We performed an association analysis using the complete adhesiome, acknowledging that not all adhesin genes are represented in the *E. coli* K-12 reference genome. This analysis yielded similar results to the whole-genome association, with significant signals for genes shared between K-12 and the STEC adhesiome (*yadK*, *ybgP*, *ybgQ*, *flu*, and *yfcS*).

**Figure 8:**
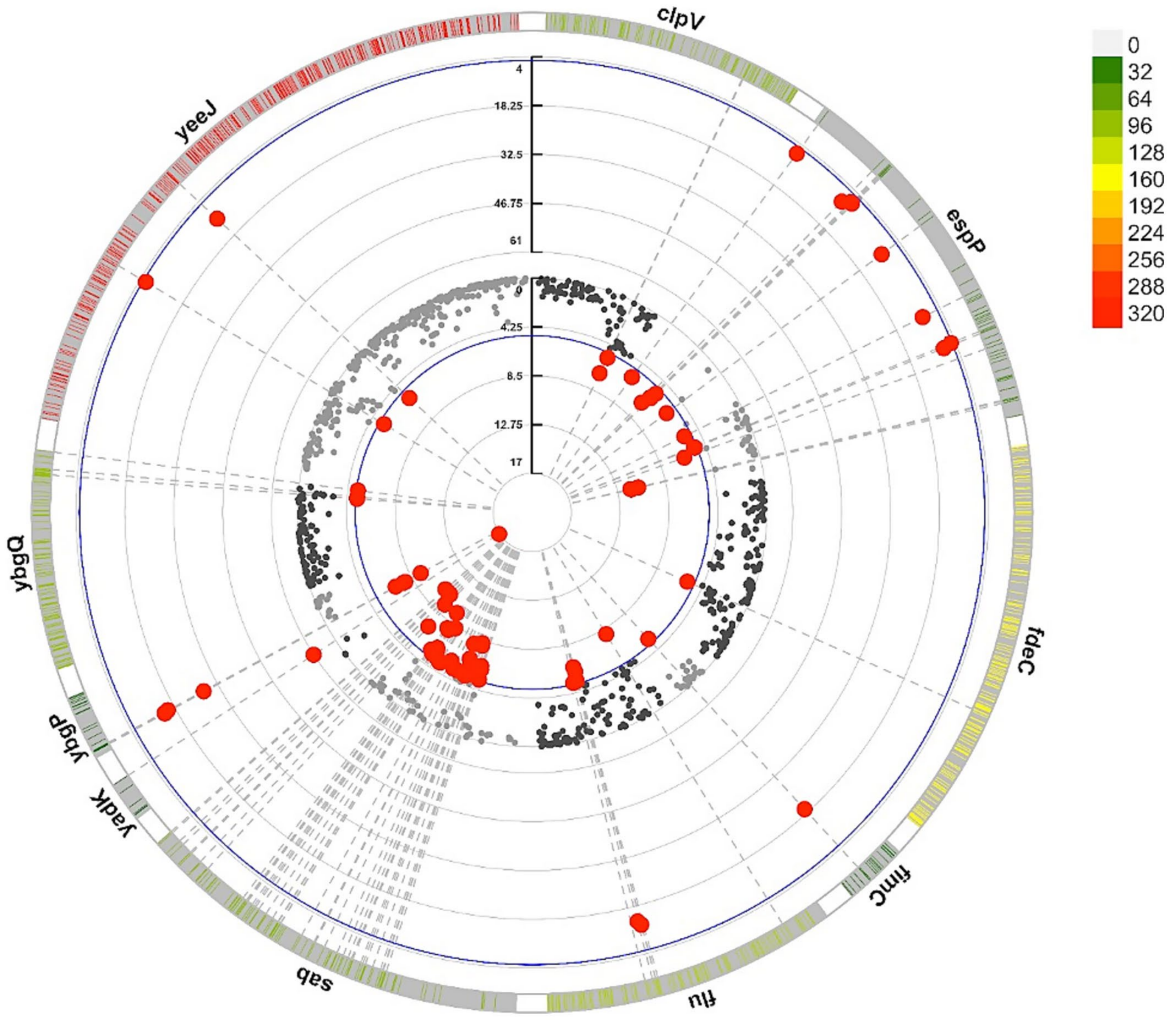
Circular representation of adhesin-associated variants in STEC genomes from cattle and human. The outermost layer shows the density of variants across adhesin genes with at least one variant significantly associated with the host, with higher color intensity indicating greater variant frequency. The middle and inner layers display the odds ratios (OR) and statistical significance (−log10(*p*)) from the GWAS, respectively, with red dots highlighting significant variants associated with cattle strains. All significant genes are listed in Supplementary Table 5.

Additionally, other adhesin genes absent from the core K-12 genome showed specific associations with the cattle host. Variants in *yeeJ*, *espP*, and *fimC* exhibited the strongest statistical associations (lowest *p*-values) and the highest OR for the cattle host (Figure 8), suggesting roles in cattle-specific colonization and adaptation under host-driven selective pressures.

Conversely, although significant, *clpV*, *ybgQ*, and *sab* displayed ORs below one, indicating stronger associations with the human host. These genes exhibited low variant density, possibly reflecting conserved functions in host interaction or membrane integrity.

Overall, these results underscore distinct adaptation strategies shaped by selective pressures, promoting STEC colonization in cattle and highlighting adhesins as potential targets for intervention strategies.

#### 3.3.4 Prediction of variant effects using the whole genome data and the adhesiome sequences

We used snpEff to predict the effects of variants identified in the GWAS analysis, utilizing the pre-compiled *E. coli* K-12 genome database. For the adhesiome sequences, a custom snpEff database was generated by annotating adhesin genes extracted from the adhesiome sequence data. Out of 433 adhesin sequences analyzed, 285 genes were successfully annotated.

The number of variants per gene was significantly higher in isolates from humans compared to cattle (4,780 vs. 3,507), suggesting a greater overall genetic diversity among human-associated STEC strains. However, the functional impact of these mutations was similar across hosts (Supplementary Table 6).

Notably, mutations with moderate-to-high predicted impacts were particularly enriched near loci associated with significant GWAS signals both in the K-12 genome and adhesiome sequences, suggesting potential adaptive selection for alternative alleles favoring host-specific colonization (Table 1). For example, an indel in *ybgQ* at position 749,777 (K-12 reference) introduced a frameshift mutation with a high predicted impact on protein function.

**Table 1:** Description of the effect of variants significantly associated with the host phenotype in the GWAS analysis of the *E. coli* K-12 strain or when considering the adhesiome sequences, and respectively with their allele frequencies.

Gene	Position	−log10(value)	Reference allele freq. in cattle	Reference allele freq. in humans	Effect of the variant	Type of change	Moderate or high in regions near the actual significant variant (100 bp)	Origin
*espP*	88	5.8	0.13	0.84	Moderate	intergenic_region	Yes	Adhesiome
*espP*	849	6.9	0.12	0.86	Low	synonymous_variant	Yes	Adhesiome
*espP*	924	6.1	0.12	0.83	Low	synonymous_variant	Yes	Adhesiome
*espP*	938	6.1	0.12	0.83	Moderate	missense_variant	Yes	Adhesiome
*espP*	948	6.1	0.12	0.83	Moderate	missense_variant	Yes	Adhesiome
*espP*	963	5.5	0.04	0.51	Low	synonymous_variant	Yes	Adhesiome
*espP*	1,625	5.9	0.18	0.92	Moderate	missense_variant	Yes	Adhesiome
*espP*	2,512	5.6	0.19	0.92	Moderate	missense_variant	Yes	Adhesiome
*espP*	2,920	5.2	0.37	0.74	Moderate	missense_variant	Yes	Adhesiome
*espP*	2,945	5.2	0.40	0.89	Moderate	missense_variant	Yes	Adhesiome
*fimC*	459	5.5	0.79	0.99	Low	synonymous_variant	Yes	Adhesiome
*flu*	1,950	5.2	0.71	0.96	Moderate	missense_variant	Yes	Adhesiome
*flu*	1,983	5.3	0.71	0.98	Low	synonymous_variant	Yes	Adhesiome
*yadK*	462	17.0	NA	0.98	Moderate	intergenic_region	Yes	Adhesiome
*ybgP*	99	7.8	0.03	0.82	Low	synonymous_variant	Yes	Adhesiome
*ybgP*	105	7.6	0.03	0.82	Low	synonymous_variant	Yes	Adhesiome
*ybgP*	111	9.4	0.05	NA	Low	synonymous_variant	Yes	Adhesiome
*ybgP*	117	6.9	0.08	NA	Low	synonymous_variant	Yes	Adhesiome
*yeeJ*	1,827	5.4	0.61	0.84	Low	synonymous_variant	Yes	Adhesiome
*yeeJ*	2,919	5.8	0.70	0.96	Low	synonymous_variant	Yes	Adhesiome
*flu*	2,072,135	5.1	0.67	0.22	Low	synonymous_variant	Yes	K-12
*yadK*	151,138	12.3	0.01	0.96	Low	synonymous_variant	Yes	K-12
*ybgP*	749,591	15.0	0.01	0.88	Low	synonymous_variant	Yes	K-12
*ybgP*	749,597	16.1	0.01	0.88	Low	synonymous_variant	Yes	K-12
*ybgQ*	749,777	15.8	0.15	0.61	High	frameshift_variant	Yes	K-12
*yfcS*	2,451,806	7.3	0.88	0.90	Low	synonymous_variant	Yes	K-12

In cattle-associated strains, alternative alleles with moderate-to-high predicted impacts were often observed at near-fixation frequencies, particularly in *yadK*, *espP*, and *ybgP*. Conversely, some genes, such as *ybgP*, exhibited moderate-to-high impact variants with low reference allele frequencies in cattle, challenging detection in GWAS analyses.

Variants occurring at intermediate frequencies and exhibiting high linkage disequilibrium with moderate-to-high impact mutations were more likely to reach significance. See Table 1 for detailed information on variants located within 100 bp of significant GWAS signals.

## 4 Discussion

Commensal bovine-adapted *E. coli* strains are considered the evolutionary precursors of diarrheagenic pathotypes, including STEC (49), with cattle-associated STEC potentially acting as a bridge to human infection (49, 50). Upon transmission, STEC must adapt to new environments and host-specific factors such as diet, hygiene, and antimicrobial exposure, all of which may shape genomic evolution (51). Therefore, identifying molecular markers that differentiate cattle- and human-associated STEC strains is essential for understanding transmission dynamics and designing targeted preventive strategies aimed at reducing bacterial carriage in livestock.

### 4.1 STEC diversity

In this study, 71 STEC strains (7.5%) were identified among the collected stool samples. This detection rate is lower than in previous reports (27, 52, 53) and may reflect several factors, including improved farm biosecurity, differences in feeding practices, and environmental conditions. Seasonal and geographical variation in STEC prevalence has been widely documented, with higher recovery rates during spring and summer (54–56). Ambient temperature, rainfall, and vector abundance have been suggested as drivers of these seasonal trends, since warmer conditions may favor STEC persistence outside the host and increase exposure opportunities. Feeding practices can also influence bacterial shedding; animals fed forage typically shed fewer STEC than grain-fed cattle (22, 57). In our study, most samples were collected during winter and early spring, a period characterized by lower temperatures and high rainfall, which may reduce environmental persistence and transmission. Moreover, in the regions analyzed, most animals were pasture-fed. Together, these factors may at least in part explain the relatively low prevalence observed. Future longitudinal studies will be required to further elucidate these factors. Importantly, despite this lower detection rate, the strains recovered are consistent with those reported in previous studies (26, 27, 58) and are representative of the STEC populations currently circulating in Chilean cattle.

Among the isolates recovered, the majority carried *stx2* subtypes, which are strongly associated with severe human disease (59). From a global perspective, *stx2* was also the predominant subtype among all analyzed STEC genomes, reinforcing concerns about the threat posed by circulating STEC strains, as Stx2 exhibits both greater cytotoxic activity and higher affinity for host ribosomes (60).

In Chile, the predominant STEC serotypes identified were O130:H11 (ST297), O185:H7 (ST2387), and O113:H21 (ST223), consistent with previous reports (26, 58), suggesting the persistence of specific strains within cattle reservoirs. In contrast, global data showed a higher prevalence of O157:H7 (ST11) and O26:H11 (ST21), along with O130:H11 (ST297). Our results underscore the considerable genomic variability in STs and serotypes among circulating STEC strains.

Notably, O130:H11 (ST297) predominated among cattle isolates, whereas O157:H7 (ST11) was more common among human isolates, consistent with global patterns (26, 61–64). O157:H7, which carries the LEE pathogenicity island and is strongly associated with the *stx2* gene, is the serotype most frequently linked to severe human disease (59, 65). Conversely, O130:H11, a LEE-negative serotype, has been implicated in sporadic outbreaks (66–68). Despite lacking the LEE locus, O130:H11 strains harbor virulence factors such as *ehxA*, *saa*, *sab*, *lpfA*, and *iha*, which may contribute to severe human disease (62, 69, 70).

Although seropathotype classification (based on ST or serotype) has historically been employed to assess the association of STEC strains with HUS and human outbreaks (71), its reliability is limited by the high genetic plasticity of STEC. The distribution of diverse serotypes across regions and hosts complicates epidemiological surveillance and strain tracking (72). Given these challenges, prioritizing molecular markers related to virulence and host adaptation over traditional serotyping could improve monitoring protocols and control strategies. This strategy would strengthen epidemiological surveillance, veterinary health measures, and public health interventions, ultimately enabling more effective and targeted control of STEC.

The high genomic variability of STEC facilitates its adaptation to diverse hosts through mechanisms such as recombination and horizontal gene transfer, impacting both virulence and transmission dynamics (49, 73, 74). However, despite this genetic plasticity, the existence of genetically homogeneous clades within specific STs suggests that certain STEC lineages have undergone niche specialization, likely driven by host- or environment-specific selective pressures. For example, at the bovine RAJ, selective pressures such as predation by bacterivorous protozoa may have favored strains encoding Stx and LEE genes, which inhibit protozoal grazing and promote persistence (50).

### 4.2 Adhesiome analyses

The evolutionary strategies of STEC may account for the greater adhesin diversity observed in human-associated strains compared to cattle-associated strains (49). Our analysis, combined with the AdhesiomeR cluster classification, confirmed that *ehaA*, *ehaG*, *stgA*-*C*, *yadL*-*N*, and *iha* were significantly more prevalent in cattle strains. EhaA, an autotransporter protein, facilitates rapid cell aggregation, biofilm formation, and adhesion to bovine RAJ epithelial cells when overexpressed in *E. coli* K-12 (19). Similarly, EhaG enhances autoaggregation, biofilm formation, and binding to collagens I–V, laminin, fibronectin, and fibrinogen (75). Although Stg adhesins have been implicated in adhesion to human and avian epithelial cells (76), their specific role in STEC gut colonization remains unclear. The Yad fimbrial autotransporter family modulates gene expression and virulence in STEC O157:H7 (77), potentially contributing to RAJ colonization (78). Additionally, Iha, a dual-function adhesin and siderophore receptor, is widely distributed among LEE-positive and LEE-negative STEC strains, and is often carried on mobile genetic elements, facilitating horizontal dissemination (11, 79, 80). Collectively, these findings suggest that cattle-adapted STEC strains rely on a relatively conserved adhesin repertoire optimized for stable colonization, biofilm formation, extracellular matrix adhesion, and iron acquisition.

In contrast, human-associated STEC strains exhibited greater diversity in adhesins, including fimbrial and non-fimbrial types, suggesting a more flexible colonization strategy adapted to heterogeneous environments. These strains showed a significantly higher representation of *eae*, *cah*, *ypjA*, and *paa* genes. Eae is a key virulence factor in LEE-positive STEC, leading to attaching and effacing lesions (81). Similarly, *cah* (calcium-binding antigen 43 homologous) is predominantly found in LEE-positive strains (82), promoting autoaggregation, biofilm formation, and persistence in STEC O157:H7 (81, 83). YpjA (homologue of EhaD) has been shown to enhance biofilm formation in STEC O157:H7, reinforcing its contribution to intestinal colonization (19). Meanwhile, *paa* (porcine attaching and effacing-associated protein) has been associated with *eae*-positive strains from both animals and human origins, suggesting a synergistic role with intimin-mediated adhesion (49, 84). These findings indicate that human-associated STEC strains rely on a broader adhesin repertoire than cattle strains, which may reflect adaptation to a more variable intestinal environment and stronger immune pressures.

Differences in adhesin gene abundance between cattle- and human-associated STEC strains align with distinct functional enrichments revealed by gene ontology analysis, supporting the notion of host-specific adaptation strategies. In cattle-associated strains, enriched biological processes point to strong selection for biofilm formation and attachment mechanisms, particularly involving fimbrial and non-fimbrial adhesins (85), autotransporter adhesins such as EhaA (86), chaperone-usher adhesins like Yad (87), and the adhesin-siderophore Iha (79). Conversely, human-associated strains exhibit significant enrichment in methylglyoxal detoxification, a response to a toxic glycolysis byproduct (88), potentially indicating a metabolic shift toward glyoxylate cycle activation. This finding suggests a potential metabolic shift toward glyoxylate cycle activation, favoring carbon conservation and survival under nutrient-limited conditions, typical of the human intestine (89). Moreover, methylglyoxal detoxification mechanisms also contribute to the neutralization of reactive oxygen species (ROS), enhancing bacterial survival against host immune defenses (90). Altogether, these observations indicate that adaptation to oxidative and metabolic stress represents a key selective force shaping the genomic and functional landscape of human-associated STEC strains.

### 4.3 GWAS

GWAS represent powerful tools for identifying genetic variants associated with bacterial adaptations, including adhesion, by linking genotype to phenotype while accounting for confounding factors such as population structure (91). Applying GWAS to pathogenic bacteria improves our understanding of virulence mechanisms and host-pathogen interactions; however, relatively few studies have explored the population structure and adaptation of STEC using this approach. Some investigations have focused on associating genomic features with pathogenic properties in STEC. For instance, Matussek et al. (92) analyzed 238 STEC genomes from patients with and without HUS to identify genetic predictors of disease severity. Their integrative approach, combining serotyping, *stx* subtyping, virulence profiling, phylogenomics, and a pangenome-wide association study (PWAS), showed that O157:H7 clade 8 strains and *stx2a* or *stx2a* + *stx2c* subtypes were strongly associated with HUS, while *stx1a* was more frequent in non-HUS cases. Virulence genes related to adherence (*eae*, *tir*, *paa*), toxins (*toxB*, *astA*), and type III secretion system proteins were enriched in HUS isolates, and hundreds of accessory genes were linked to severe disease, including adhesins (*yfcP*, *yehD*, *elfG*, *sfmA*) and regulators, although many encoded hypothetical proteins. The authors concluded that severe human disease results from the interplay between canonical virulence determinants, accessory genetic elements, and host–pathogen interactions. Similarly, Peroutka-Bigus et al. (93) compared genomic and phenotypic features of three human outbreak-associated and one cattle-derived STEC O157:H7 isolates to assess host adaptation. Despite differences in virulence gene expression, adherence, and Stx production among outbreak isolates, no significant differences were detected in cattle colonization or shedding compared with the cattle-associated strain. This highlights that genomic and phenotypic variation in STEC O157:H7 does not necessarily correspond to host-specific adaptation, emphasizing that host specificity cannot be inferred solely from genetic or phenotypic traits. More recently, Espadinha et al. (94) performed a PWAS of 531 STEC isolates, identifying associations between the development of HUS and the presence of *stx2a*, *stx1a* + *stx2a*, or *stx1a* + *stx2c*, as well as the co-occurrence of genes such as *ygiW* (stress-induced protein) with group_5720 (transcriptional regulation) and *pfkA* (6-phosphofructokinase-1) with *fieF* (Zn^2+^/Fe^2+^/Cd^2+^ efflux transporter), among other epidemiological factors. In the same context, Marques Da Silva et al. (95) explored the genomic determinants of cattle colonization by comparing the genomes of STEC O22:H8 and O157:H7 strains. They identified 28 virulence-associated genes unique to O22:H8, primarily involved in adherence (e.g., *cfaA*, *cfaB*, *cfaC*, *cfaD*/*cfaE*, *sisA*, *lesP*, *hes*, *pagC*, *tpsA*, and *tpsB*), autotransporters (*ag43*), and invasion (*tia*). These findings highlight the complexity of adhesin gene distribution and function in STEC, reinforcing the need for further research to elucidate the genetic basis of host-specific colonization.

Beyond the host-specific differences between cattle- and human-associated STEC strains, our GWAS comparing these isolates with the non-pathogenic *E. coli* K-12 genome provides additional evidence for host-driven selection, highlighting potential adaptive traits that distinguish STEC from commensal *E. coli* strains. This analysis revealed significant enrichment of trehalose transport and protein-phosphocysteine-trehalose phosphotransferase system activities, associated with the *bglF* and *ascF* genes. It is well established that *E. coli* can use trehalose as an alternative carbon source and synthesize it intracellularly to counteract osmotic stress by stabilizing membrane integrity (96). The BglF and AscF proteins, key components of the phosphotransferase system, mediate the transport and phosphorylation of *β*-glucosides, including trehalose, thus regulating its metabolism and contributing to the bacterial stress response (97). These metabolic adaptations may enhance the resilience of STEC strains to osmotic and environmental stresses encountered in the bovine gastrointestinal environment.

Our GWAS comparing the adhesiome of cattle- and human-associated STEC with *E. coli* K-12 revealed distinct host-specific adhesion strategies. In cattle strains, *yadK* and *ybgP* were significantly associated. YadK, a chaperone-usher adhesin, enhances acid stress resistance, biofilm formation, and epithelial attachment, promoting persistence in the bovine gastrointestinal tract (98, 99). YbgP facilitates adhesion to epithelial and abiotic surfaces, promoting environmental persistence (87, 100). In contrast, human-associated strains exhibited significant enrichment of *ybgQ*, *yfcS*, and *flu*. *ybgQ* encodes the usher protein necessary for YbgP fimbriae assembly, suggesting a role in epithelial colonization (101). YfcS enhances biofilm formation and bacterial aggregation (87, 100), while *flu* (also known as *agn43*), an autotransporter adhesin, promotes microcolony formation, biofilm stability, and immune evasion in both LEE-positive and LEE-negative STEC strains (11, 102). These findings suggest that cattle-associated STEC strains prioritize adhesion mechanisms suited for long-term gut colonization, whereas human-associated strains exhibit a broader adhesin repertoire, likely reflecting selective pressures favoring host invasion, immune evasion, and adaptation to the intestinal niche.

Notable differences emerged when analyzing the complete adhesiome of cattle- and human-associated STEC strains compared to the analysis based solely on the *E. coli* K-12 genome. In cattle-associated strains, *yeeJ*, *espP*, and *fimC* exhibited the highest significance values and OR, suggesting a prominent role in host-specific colonization and adaptation, likely driven by selective pressures within the bovine gastrointestinal tract. *yeeJ* encodes an autotransporter protein with structural similarity to intimin, facilitating biofilm formation and enhancing STEC O157:H7 binding to eukaryotic cells (103, 104). EspP, a serine protease autotransporter of *Enterobacteriaceae* family, encoded on STEC virulence plasmids, promotes adhesion to bovine rectal epithelial cells, intestinal colonization, and supports biofilm formation and HeLa cell adherence (105, 106). Its proteolytic activity targets extracellular matrix and mucus proteins, facilitating tissue penetration and receptor exposure for other adhesins. *fimC*, a key component of the *fim* operon, plays a critical role in the assembly of type 1 fimbriae, which promote epithelial adhesion and biofilm formation in *E. coli*. In STEC O157:H7, type 1 fimbriae have been implicated in RAJ cell colonization, highlighting their potential contribution to cattle adaptation and long-term persistence (107, 108).

Conversely, *clpV*, *ybgQ*, and *sab* exhibited a highly significant OR below one, suggesting their association with human-host adaptation. ClpV, a cytosolic ATPase and essential component of the type VI secretion system (T6SS), facilitates bacterial competition by delivering effector proteins—such as peptidoglycan hydrolases, phospholipases, and DNases—into target cells (109, 110). In the STEC O157:H7 strain EDL933, ClpV mediates the translocation of catalase into macrophages, promoting immune evasion; notably, deletion of *clpV* reduces lethality in murine infection models (111). Furthermore, ClpV has been associated with HUS-producing STEC strains, supporting its potential role in virulence (112, 113), although its precise contribution to intestinal colonization remains unclear. Similarly, Sab, a plasmid-encoded autotransporter, enhances adherence to human epithelial cells and biofilm formation in LEE-negative STEC strains (114), potentially facilitating intestinal colonization and persistence.

In addition to the gene presence/absence patterns identified through GWAS, variant analysis offered deeper insights into host-specific selective pressures shaping adhesin functionality. Human-associated STEC exhibited greater genetic diversity, although the predicted functional impacts on adhesins were largely conserved across hosts. A key observation was the presence of SNPs with moderate-to-high effects on protein sequences. In particular, given *ybgQ*’s role in adhesion and outer membrane integrity, structural disruptions caused by SNPs may significantly affect bacterial adherence and survival within the human gut. Conversely, cattle-associated strains exhibited alternative mutations with moderate-to-high effects, especially in *yadK*, *espP*, and *ybgP*, where near-fixation frequencies suggest strong host-driven selection favoring these variants for bovine colonization. Notably, different *espP* alleles exhibit distinct biological activities: EspPα and EspPγ are secreted and enzymatically active, whereas EspPβ and EspPδ display reduced or absent proteolytic function (115). Among them, EspPγ specifically cleaves pepsin and coagulation factor V in humans (116), while EspPα is more frequently found in human isolates. In contrast, other EspP variants are predominantly associated with animal reservoirs and environmental sources (117). These host-driven selective pressures likely induce functional modifications that may either enhance or attenuate virulence. Further experimental studies, including *in vitro* and *in vivo* infection models, are necessary to clarify the impacts of these alternative variants on colonization efficiency and adhesin functionality.

A potential limitation of our GWAS is the uneven geographic distribution of publicly available STEC genomes, with an over-representation of isolates from countries such as Chile, Germany, and France. However, our analyses were focused on host origin rather than country of isolation, and GWAS methods corrected for population stratification were applied to mitigate this potential bias. In addition, the dataset encompassed diverse serotypes and lineages, which supports the robustness of the host-specific associations identified.

Targeting adhesins in STEC offers a promising avenue for developing effective intervention strategies. Given the essential role of adhesins in host colonization, strategies such as anti-adhesin antibodies, competitive inhibitors, or adhesin-based vaccines could significantly reduce bacterial adherence and gut colonization, ultimately lowering transmission risk. These approaches could be particularly beneficial in pre-harvest control programs designed to decrease STEC carriage in cattle. Future research should focus on identifying adhesins with high conservation across STEC strains and evaluating their potential as preventive targets, including efficacy assessments through animal model studies.

## 5 Conclusion

This study provides new insights into the genetic diversity and functional roles of adhesins in STEC host adaptation. We identified adhesin-encoding genes strongly associated with cattle strains (e.g., *yadK*, *espP*, *fimC*) and others in human-associated strains (*clpV*, *ybgQ*, *sab*) whose presence and polymorphisms may reflect host-specific selective pressures. Importantly, these genes are not only markers of host origin but also have potential functional implications, where cattle-associated strains may display enhanced colonization and persistence at the bovine RAJ, while human-associated strains may have facilitated immune evasion and adhesion to epithelial cells, increasing their virulence in the human host. However, their precise roles in host colonization require further investigation, particularly to evaluate their potential as intervention targets. Future functional studies, including adhesion assays and transcriptomic profiling under host-mimicking conditions, will be crucial to elucidate their contribution to bacterial fitness, biofilm dynamics, and immune evasion strategies. Moreover, variant analysis revealed greater genetic diversity in adhesin genes among human-associated strains, although functional effects remained comparable across hosts, suggesting selective constraints that preserve key adhesion mechanisms. High-impact mutations should be further examined through protein structure modeling and functional assays to assess their influence on adhesion efficiency and pathogenicity. Collectively, such studies may contribute to the identification of conserved adhesin targets for vaccine development and other intervention strategies aimed at reducing STEC transmission at the livestock level.

## Data Availability

The whole-genome sequences of STEC strains that support the findings of this study are openly available in GenBank at https://www.ncbi.nlm.nih.gov/genbank/, reference number PRJNA656305.

## Footnotes

^1^ www.ncbi.nlm.nih.gov/genbank/

^2^ https://github.com/bbuchfink/diamond

^3^ https://github.com/chklovski/CheckM2/blob/main/README.md

^4^ https://github.com/tseemann/abricate

^5^ https://github.com/tseemann/mlst

^6^ https://cge.food.dtu.dk/services/SerotypeFinder/

^7^ https://github.com/ksidorczuk/adhesiomeR.git

^8^ https://adhesiomer.quadram.ac.uk/app/adhesiomeR
