# Supplementary material for: Comparative genomics of Shiga toxin-producing Escherichia coli reveals host-specific adhesiome adaptations in humans and cattle
Source: Front Vet Sci. 2025 Oct 9;12:1639243. doi: 10.3389/fvets.2025.1639243 (PMC12547504; doi:10.3389/fvets.2025.1639243)
Supplement: Supplementary file 1 [file Image_1.pdf]

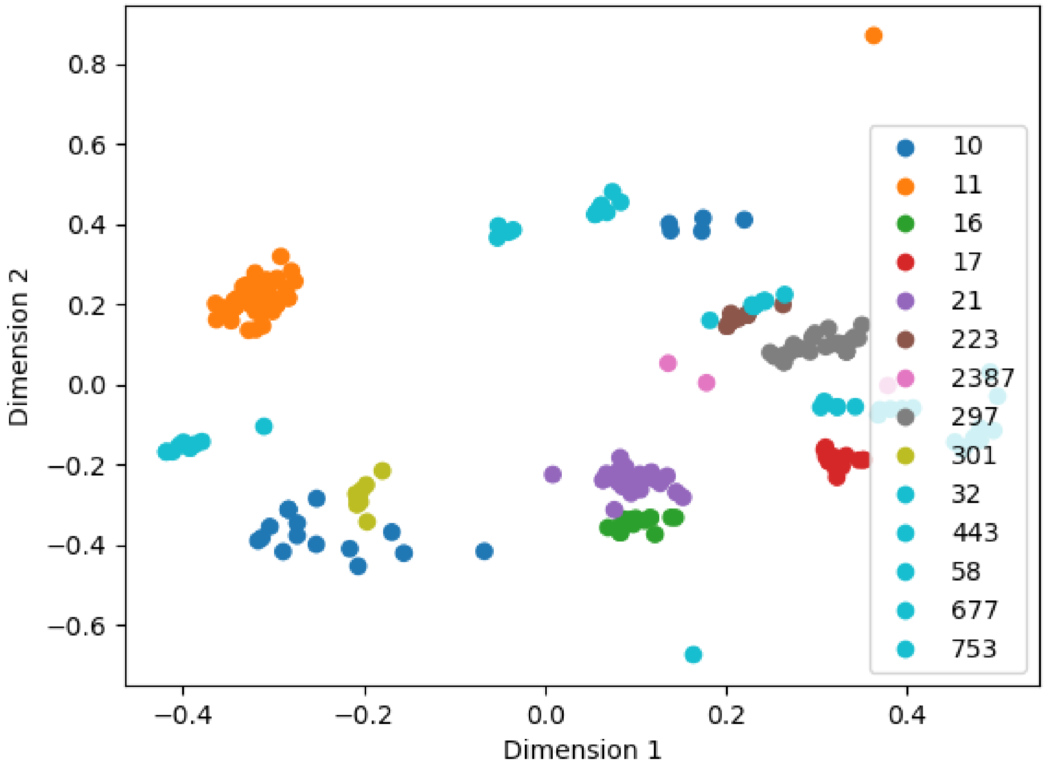

Supplementary Figure 1: Whole genome sequence multidimensional scaling analysis according to the MLST genotypes. Each MLST group is shown with different colors
